# Distribution of case volumes in surgery: an analysis of the British Spine Registry

**DOI:** 10.1136/bmjsit-2023-000202

**Published:** 2024-03-22

**Authors:** Chan Hee Koh, William Muirhead, Danyal Zaman Khan, Hugo Layard Horsfall, George Prezerakos, Parag Sayal, Hani J Marcus

**Affiliations:** 1 Victor Horsley Department of Neurosurgery, National Hospital for Neurology and Neurosurgery, London, UK; 2 Wellcome / EPSRC Centre for Interventional and Surgical Sciences, University College London, London, UK; 3 Neurosciences Institute, Cleveland Clinic London, London, UK; 4 UCL Queen Square Institute of Neurology, London, UK

**Keywords:** Health Services Research, Health Policy, Health Care Quality, Access, and Evaluation, Learning Curve

## Abstract

**Objectives:**

To characterize the distribution of case volumes within a surgical field.

**Design:**

An analysis of British Spine Registry.

**Setting:**

295 centers in England that conducted at least one spinal operation either within the NHS or private settings between 1 May 2016 and 27 February 2021.

**Participants:**

644 surgeons.

**Main outcome measures:**

Mathematical descriptions of distributions of cases among surgeons and the extent of workforce-level case-volume concentration as a surrogate marker.

**Results:**

There were wide variations in monthly caseloads between surgeons, ranging from 0 to average monthly high of 81.8 cases. The curves showed that 37.7% of surgeons were required to perform 80% of all spinal operations, which is substantially less than in fields outside of healthcare.

With the COVID-19 pandemic, the case volumes of surgeons with the highest volumes dropped dramatically, whereas those with the lowest case numbers remained nearly unchanged. This, along with the relatively low level of case-volume concentration within spinal surgery, may indicate an inevitability of at least some level of surgical care being provided by the relatively lower volume surgeons.

**Conclusions:**

While there is a reasonable degree of workforce-level case volume concentration within spinal surgery, with high volume spinal surgeons providing a large proportion of care, it is not clear whether a further concentration of case volumes into those few hands is possible or desirable.

**WHAT IS ALREADY KNOWN ON THIS TOPIC**

- It is widely believed that within a given field, 80% of work is done by 20% of ‘highly productive’ people. However, it is unclear the extent to which this applies within surgery or healthcare in general.

**WHAT THIS STUDY ADDS**

- In British spinal surgery, it requires 38% of surgeons to perform 80% of operations, which is substantially less unequal than in fields outside of healthcare.

**HOW THIS STUDY MIGHT AFFECT RESEARCH, PRACTICE OR POLICY**

- Increased concentration of case volumes in individual surgeons has been shown to improve outcomes, and as such, there may be scope for further workforce-level specialization and concentration of case volumes. However, it is not clear that this is possible or necessarily desirable, and other methods of overcoming the potential disadvantages or relatively lower case volumes may need to be considered.

## Introduction

It is a commonly espoused principle that the majority of workload and productivity is concentrated in the hands of a productive or specialized few. Rules such as Pareto principle (‘80% of work is done by 20% of people’) have been shown to hold true in fields such as finance and economics.^1^ Another well-known law called Price’s law (‘half of the total output is produced by square root of the total contributors’) was formulated in scientometrics. A related law in scientometrics is Lotka’s (inverse power) law, which states that the number of people who contribute a certain number of papers is proportional to the inverse power of the number of papers.

Although the validity of Price’s law is unclear,^2^ the validity of Lotka’s law within scientometrics has been well established,^3 4^ as well as in many other contexts including word-frequency distribution and population of US cities.^5^ While these laws have captured popular imagination and is commonly assumed to approximate the truth in multiple fields, there are examples of deviations away from these expectations, such as firm sizes and length of days in a relationship.^5 6^ It is also not clear whether these hold true within surgery or healthcare. For example, length of hospital stay and operating room turn-over time can be well modeled by a power law distribution.^7 8^ There have been some attempts to describe distribution of operations within surgery, particularly in the context of global health.^9^ However, there are no mathematical equivalents such as Price’s law within the field surgery and case-volume distributions.

Here, we present the first attempt at providing a mathematical description of the distribution of labor within medicine and surgery, using British spinal surgery as an exemplar. We analyzed the distribution of case volumes within spinal surgery. We subsequently investigated the effects of COVID-19 and the resulting health policies on this distribution.

## Methods

The paper is presented in accordance with the Strengthening the Reporting of Observational Studies in Epidemiology (STROBE) statement for cross-sectional studies.^10^

### Data acquisition and cleaning

The British Spine Registry (BSR) was established in May 2012 by the British Association of Spinal Surgeons to with the aim of collecting information on all spinal surgeries, performed in the National Health Service (NHS) and in the private sector, throughout the UK.^11^ To this extent, the ‘Best Practice Tariff’ was introduced in 2019 by NHS England and NHS Improvement to improve compliance with data entry.^12^ The target ascertainment rate is 50%, which was achieved by 55% of units in the first quarter after implementation, with plans to increase to 80% in the near future.

Data from the BSR from inception to 27 February 2021 were acquired. The information acquired was ‘unit’, ‘consultant in charge’ and ‘procedure completed date’. Each surgeon is assigned a unique entry in the database, which is consistent regardless of the location of surgery.

Data prior to 1 May 2016 were excluded, as the completion rate of the fields ‘unit’ and ‘consultant in charge’ were poor before that date (online supplemental table 1 and 2). Given the introduction of ‘Best Practice Tariff’ in April 2019, and the COVID-19 lockdown in March 2020, we derived the final coefficient estimates from data entered between these two dates.

10.1136/bmjsit-2023-000202.supp1 Supplementary data

The BSR does not hold information on whether the individual surgeons are actively practicing, temporarily or permanently inactive. Therefore, active spinal surgeons were defined as those who had performed at least one spinal operation from April 2019 onwards, which allowed an estimation of the number of surgeons who had performed no spinal operations within each month.

### Data processing

Separate analyses were conducted by consultant and by hospital unit. From here on, the surgeon is defined as consultant in charge.

For testing of Lotka’s law and alternative formulations, a frequency table of ***Y*** number of surgeons doing ***X*** number of operations grouped by month was created.

For testing of Price’s law and alternative formulations, the data were ordered by the number of cases performed by each surgeon. The cumulative count of surgeons and the cumulative proportion of operations for each surgeon were calculated.

### Data analysis

All curve fitting was done using linear and non-linear mixed-effects regressions using *R* statistical programming V.4.1.0 and package *nlme* V.3.1.^13 14^ The coefficients of curves were set as fixed effects, and the year and month of operation as nested random effects. The fitted curves and the residuals were visualized.

The fit provided by the curves was assessed using Akaike information criterion (AIC), which gives numerical measures of the quality of each model by evaluating goodness-of-fit while penalizing model complexity.^15^ Penalizing model complexity is necessary as increasing model complexity leads to overfitting, diminishing real-world performance on external data in spite of the apparent improvement in goodness-of-fit on the observed dataset. AIC was preferred to Bayesian information criterion, which more heavily penalizes model complexity, to prioritize prediction accuracy over model sparsity.

Data visualization was done using the ggplot package.^16^ The following important dates are indicated on the plots:

- April 2019—Introduction of “Best Practice Tariff” for the BSR to encourage data entry.
- April 2020—First British COVID-19 lockdown on 25 March 2020.
- November 2020—Second British COVID-19 lockdown on 5 November 2020.
- January 2021—Third British COVID-19 lockdown on 5 January 2021.

Geographical data were plotted using *sf* V.0.9,^17^ *rgeos* V.0.5^18^ and *rgdal* V.1.5.^19^ The boundaries of NHS England regional teams (London, South East, South West, East of England, Midlands, North East and Yorkshire, North West) in April 2020 were obtained from the Office for National Statistics.^20^

## Results

### Summary of the BSR data

Between 1 May 2016 and 27 February 2021, there were a total of 152 066 operations logged on the BSR. There was a median of 2005 (IQR: 1536–2268) operations each month, ranging from 411 operations in April 2020 to 3621 operations in October 2019 (online supplemental figure 1A).

There were a total of 643 surgeons performing at least 1 spinal surgery during that period, with a median of 231 (IQR: 176–308.2) operating surgeons per month, ranging from 111 surgeons in July 2016 to 452 surgeons in October 2019 (online supplemental figure 1B). There were 285 units that performed at least one spinal operation, with a monthly median of 138.5 (IQR: 119.8–154), ranging from 61 units in April 2020 to 189 units in February 2020 (online supplemental figure 1C). 144 of these units were NHS units, and 141 were independent units. 340/643 surgeons (52.9%) performed spinal surgeries in more than 1 unit, with 4/643 surgeons (0.006%) performing spinal operations in 7 different units.

There were fewer records in the earlier years, which is likely to reflect the relatively poorer (although slowly improving) compliance in the earlier years. With the introduction of the ‘Best Practice Tariff’, which in effect mandated data entry into the registry, a subsequent stabilization in the number of records can be observed (online supplemental figure 1).

### The majority of surgeons and units have low volumes of spinal operations

**Figure 1:**
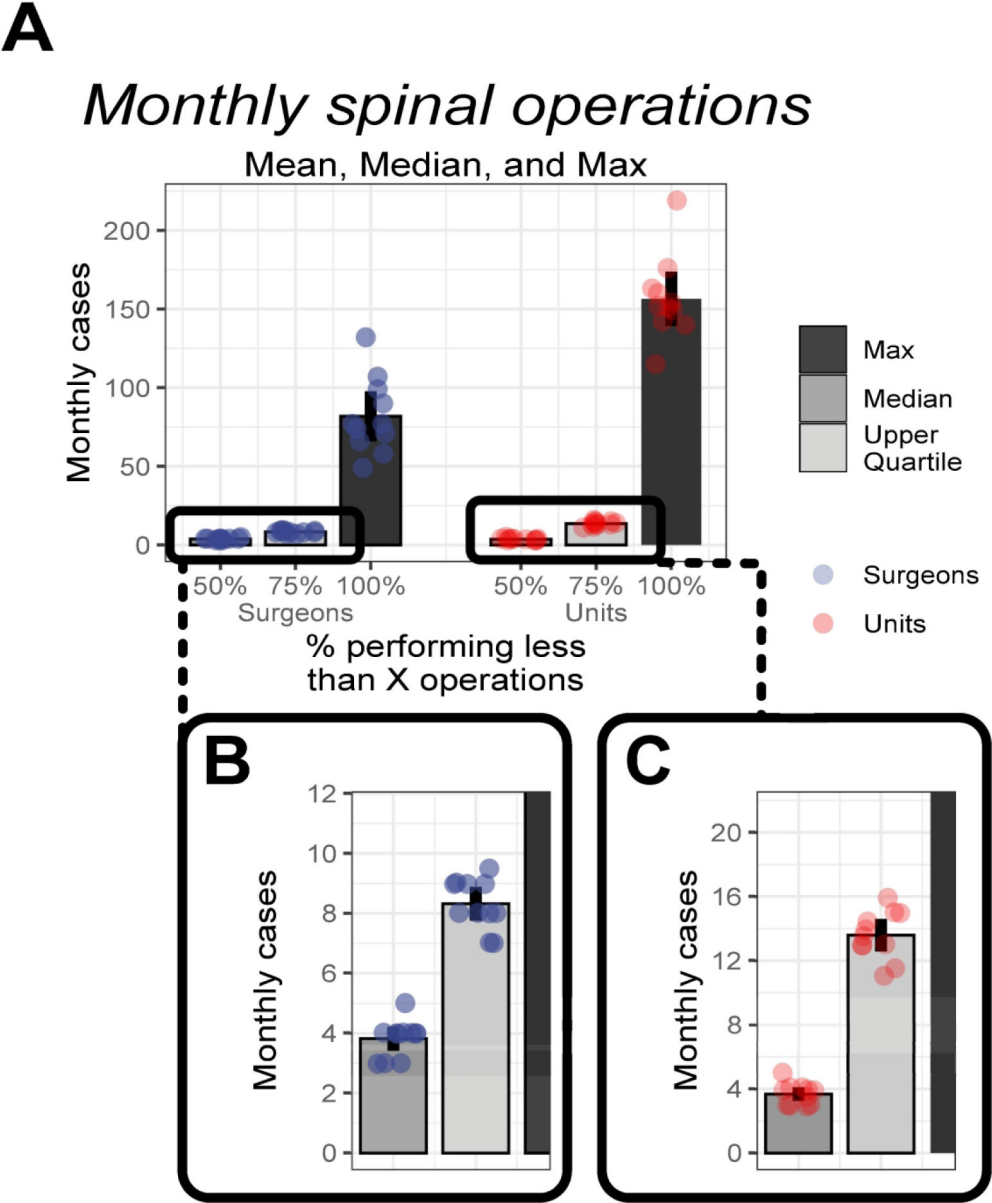
Variations in monthly case volumes. (A) The mean of the monthly medians, monthly 75th centiles, and monthly maximums of surgeons and units. (B) A zoomed plot of monthly median and upper quartile for surgeons. (C) A zoomed plot of monthly median and upper quartile for units. Bars denote the mean. The error bars denote the 95% CIs.

Between April 2019 (introduction of ‘Best Practice Tariff’) and March 2020 (COVID-19 lockdown), there were wide variations in monthly case volumes of individual surgeons between a range of 0 and 81.8 (95% CI 65.9 to 97.8) cases per month (figure 1A). There was a similar variation in the case volumes of individual units between a range of 0 and 158.6 (95% CI 141.3 to 175.6; figure 1A).

The vast majority of surgeons performed less than 10 spinal operations each month, with the average of the median monthly figure being 3.8 (95% CI 3.4 to 4.2; figure 1B). A sizeable majority of units performed less than 15 spinal operations each month, with the average of the median monthly figure being 7.8 (95% CI 3.2 to 4.1; figure 1C).

### Describing the distribution of case volumes

**Figure 2:**
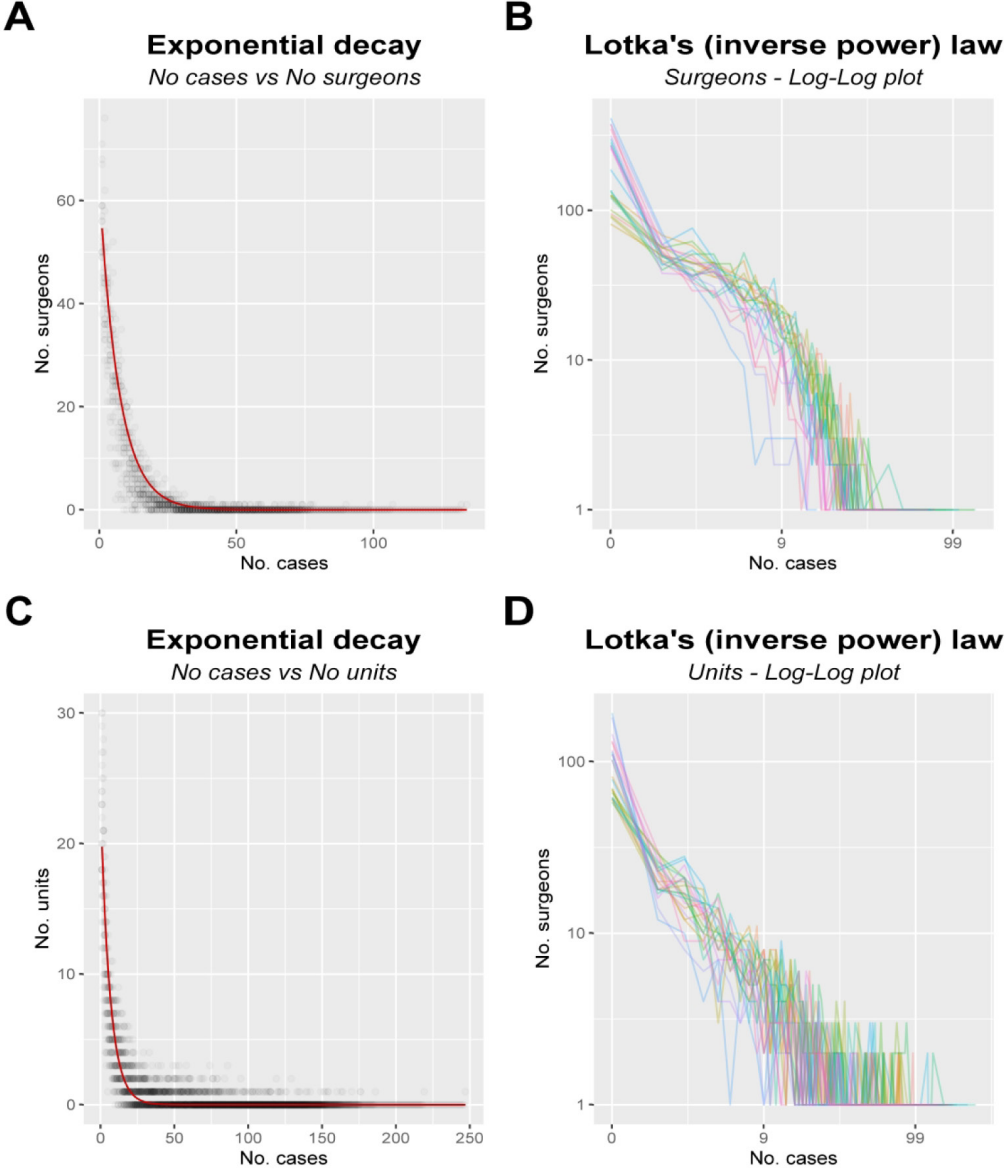
Distribution of labor in British spinal surgery. (A) The distribution of a number of surgeons who perform a certain number of operations. (B) A log-log plot, with both axes on a long scale, for visual inspection for an inverse power law. (C) The distribution of number of units performing a given number of operations. (D) A log-log plot for the data in (C).

Similar to Lotka’s law, we found that the most frequent number of monthly operations by spinal surgeons and spinal units was 0, with increasingly smaller numbers of surgeons and units performing larger number of operations (figure 2A–D).

With respect to distribution of monthly case volumes among spinal surgeons, a visual inspection of the log-log plot showed a clearly non-linear trend (figure 2B). This suggests that the distribution of of case volumes does not respect an inverse power distribution. A quantitive analysis showed that an exponential decay is a better fit for this distribution as measured by the AIC (figure 2A).

The distribution of monthly case volumes among units showed a more linear relationship on the visual inspection of the log-log plot (figure 2D). Even in this case, however, an exponential decay was found to be a better description of the data (figure 2C).

### Lesser concentration of case volumes within spinal surgery compared with other fields

Pareto’s principle and Price’s law are two well-described principles, describing the concentration of productivity in the hands of a few ‘superstars’, and which have been shown to hold true in multiple fields.

**Figure 3:**
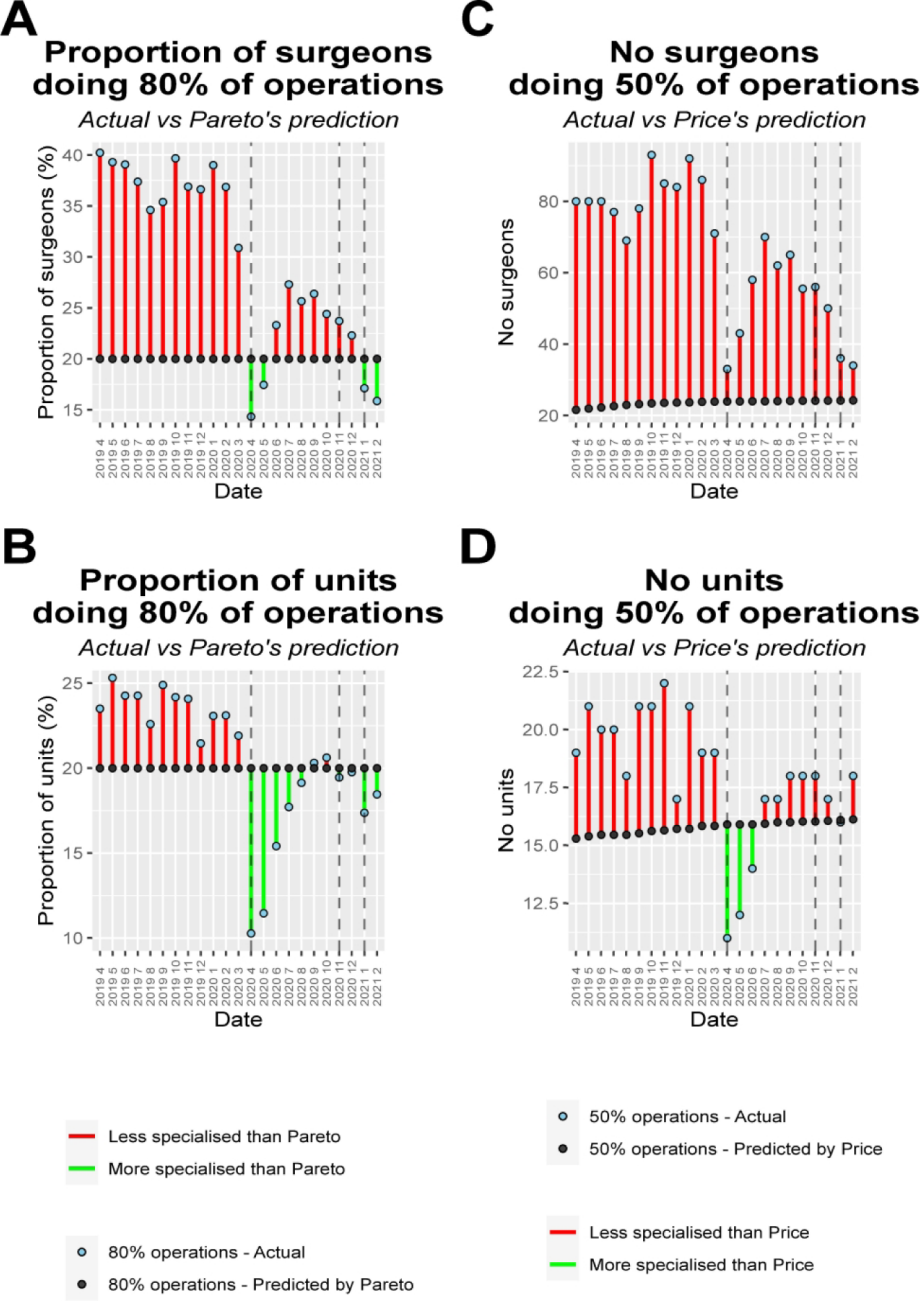
Pareto’s principle and Price’s law versus reality. (A) Comparing Pareto’s predictions with reality with regard to the proportion and numbers of surgeons performing 80% of all operations. (B) Comparing Pareto’s predictions with reality with regard to the proportion of units performing 80% of all operations. (C) Comparing Price’s predictions with reality with regard to the nth root of total number of surgeons and the numbers of surgeons performing 50% of all operations. (D) Comparing Price’s predictions with reality with regard to the nth root of total number of units and the numbers of units performing 50% of all operations.

Within spinal surgery, we found that the Pareto’s principles and Price’s law far overpredict the number of operations performed by the few highest volume surgeons (figure 3A–D). This was true for every single month from the beginning of the dataset in 2016 with the very few exceptions in the early period when data entry was less complete (figure 3D). Our data showed that in fact:

1. 80% of operations were performed by 37.7% of surgeons (95% CI 36.5% to 39.0) as opposed to 20% of surgeons as predicted by Pareto’s principle (figure 3A). 50% of operations were performed by 15.6% of surgeons (95% CI 14.8% to 16.4%).
2. Half of all operations were performed by the 1.42th root of the total number of surgeons (95% CI 1.41 to 1.44). This is in contrast to 2nd root (ie, square root) as predicted by Price’s law (figure 3C).

The distribution among spinal units was also more equal than would be expected from other fields, although somewhat more in line with Pareto’s principle and Price’s than the distribution among spinal surgeons.

1. 80% of operations were performed by 23.7% of units (95% CI 23.0% to 24.4%), which is closer to 20% as predicted by Pareto’s principle (figure 3B). 50% of operations were performed by 8.2% of units (95% CI 7.8% to 8.7%).
2. Half of all operations were performed by the 1.84th root of the total number of units (95% CI 1.80 to 1.87). This is close to the Price’s law that half of operations are performed by the second root (figure 3C).

### The impact of COVID-19 on case distributions

There were dramatic changes in the concentration of case volumes with the COVID-19 pandemic. The changes were most pronounced with the first national lockdown, where case-volume concentration among spinal surgeons and spinal units increased, going from being far below Pareto’s and Price’s predictions, to being far greater in most cases (figure 3A–D).

**Figure 4:**
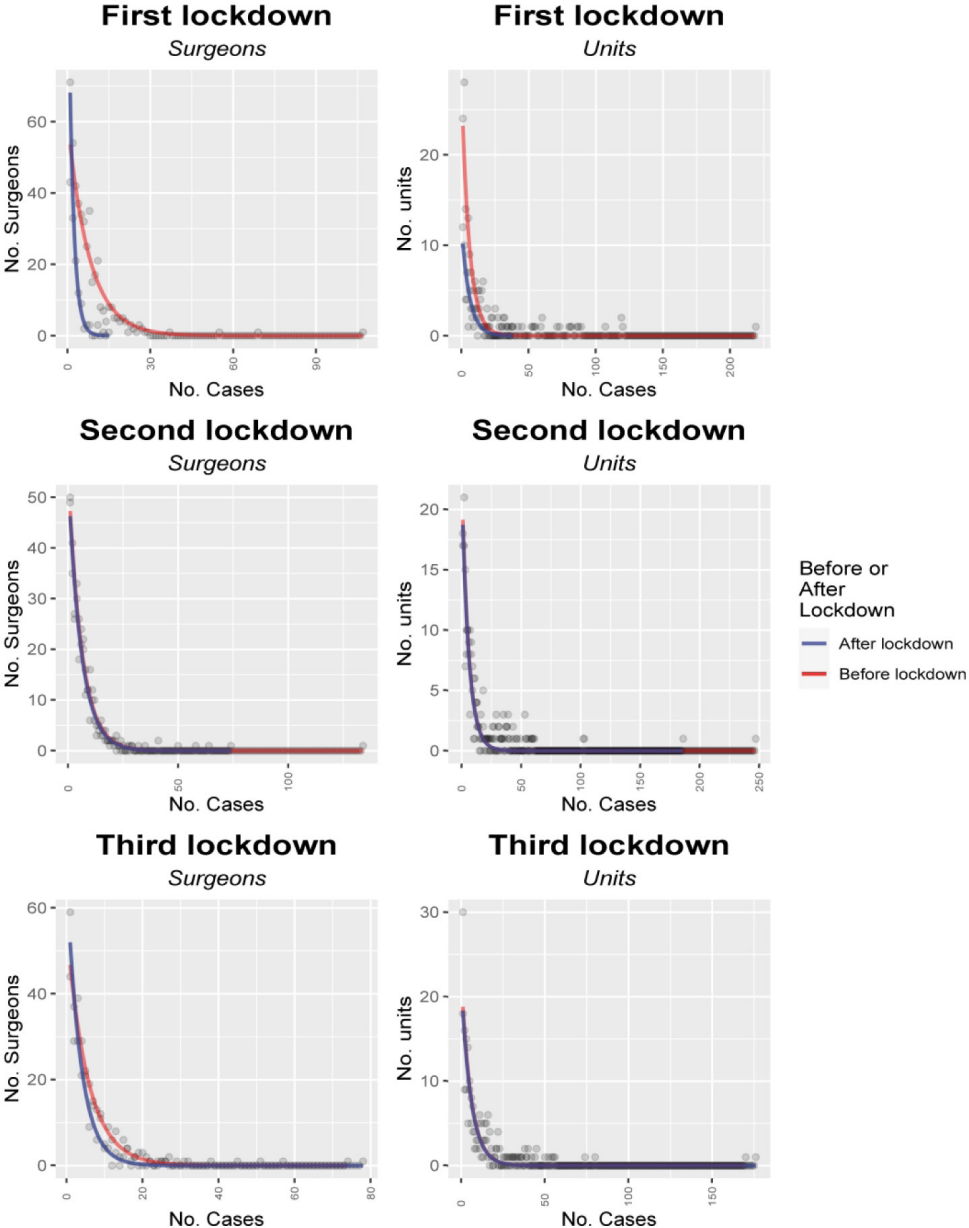
Changes in distribution with COVID-19.

**Figure 5:**
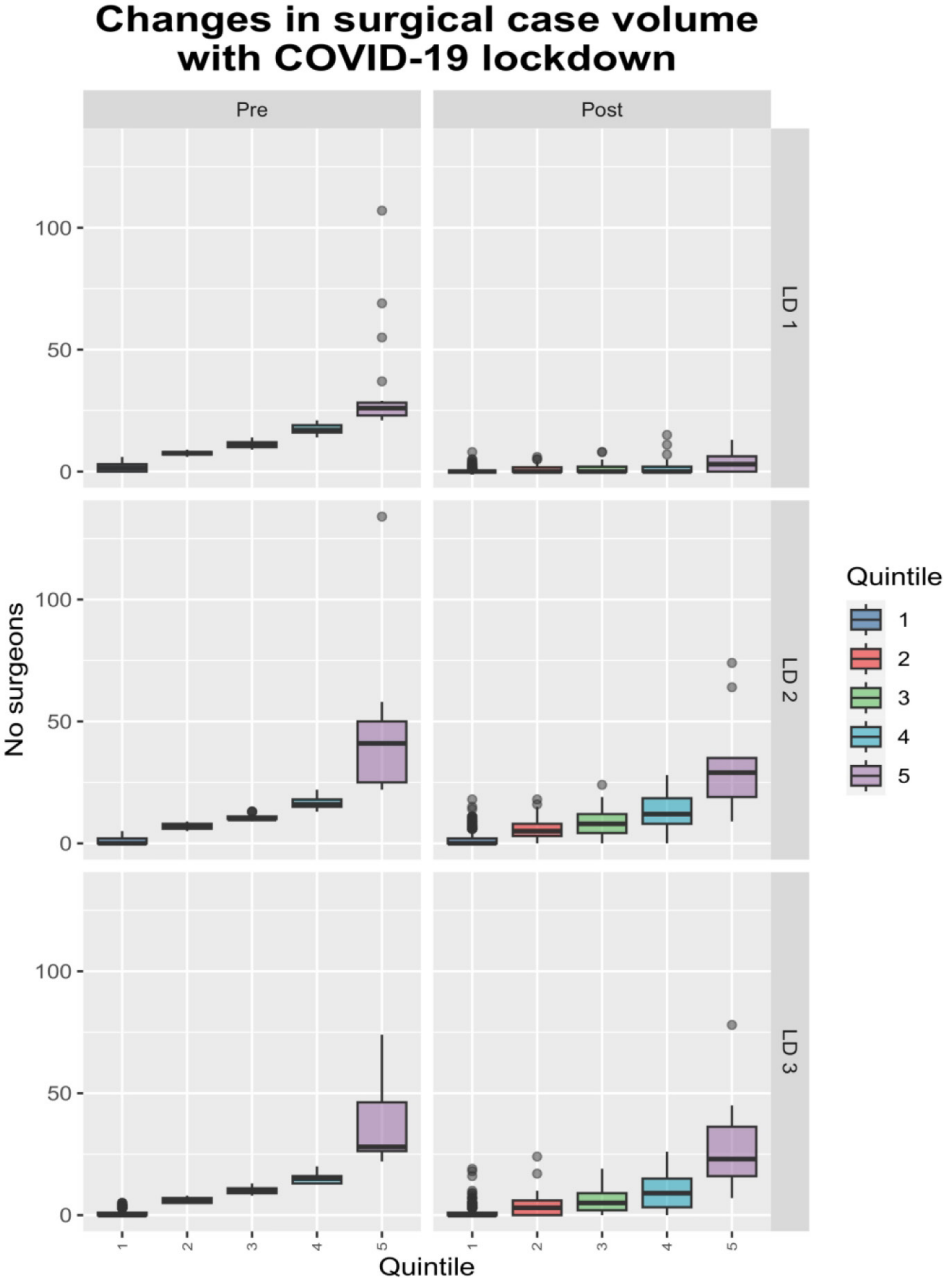
Changes in case volumes with COVID-19, grouped by monthly case volumes of surgeons and units. The individual surgeons and units are grouped by the monthly case numbers and tracked across the time periods above, with the first group being the largest comprising the largest number of lowest-volume surgeons/units performing the first 20% of all spinal operations. The second group is then the second largest, comprising the next group of low volume surgeons contributing to 20% of operations, and so on, until the final group comprises the small number of highest volume surgeons contributing to 20% of all operations. Whiskers indicate range (with outliers indicated by points), box the IQR and the middle line the median.

Further inspection of the data showed that although there was a decrease in high-volume surgical activity, there was also a substantial increase in the number of surgeons performing zero operations during these periods (figure 4). Moreover, it was the surgeons with the highest case volumes that experienced the greatest drop in the number of cases, whereas the group of surgeons with the lowest number of cases experienced far smaller changes in their overall case volumes (figure 5).

The changes were less dramatic during the subsequent lockdowns partly due to the persistence of the changes in the distribution, but also the less stringent nature of these subsequent lockdowns.

## Discussion

### Summary of findings

Pareto’s principle, Price’s law and Lotka’s law are often used to encapsulate the idea that a small group contribute disproportionately to a large proportion of the output. The empirical data within the field of surgery (with British spinal surgery as exemplar) does not support the specific or the generalized mathematical formulations of these doctrines. However, the general principle holds true that (1) the contributions of a minority within this field far exceed those of the majority (figure 1) and that (2) there is an unequal distribution of contributions within this field such that a minority of the workforce contribute to the majority of the output (figure 3).

The exponential curves, that better describe the empirical data presented here, also share with the above doctrines in that the greatest number of spinal surgeons performed only one or no case in any given month (figure 2). This suggests that while there are a number of spinal subspecialists (as indicated by higher case volumes), there is still a large proportion of spinal operations performed by non-subspecialist surgeons.

When compared with observations in non-medical fields as described by Pareto’s principle and Price’s law, there was substantially less concentration of case volumes among spinal surgeons than might have been expected (figure 3A,C). 80% of spinal operations were performed by 37.7% of surgeons, rather than by 20% as described in other fields by Pareto’s principle.

There were evident increases in the case-volume concentration with the COVID-19 lockdowns (figure 3), when there were marked reductions in case volumes and cancellations in elective spinal surgery (figure 4).^21^ The remaining (predominantly emergent) caseload was not evenly redistributed among surgeons, with the number of operations by the lowest volume surgeons being relatively unchanged in comparison the disproportionate reductions in the numbers by highest-volume surgeons (figure 5), along with little change in the total number of surgeons performing only one operation. The marked reduced demand could be met by a small number of lower-volume surgeons resulting in increased case-volume concentration, rather than requiring the greater numbers of medium-volume and high-volume surgeons (figure 4). However, the fact that the reduced, predominantly emergent, post-lockdown caseload was not proportionately redistributed to high-volume surgeons relative to their usual activity, in spite of the apparent freeing up of capacity, suggests that there may be obstacles to providing a complete concentration of case volumes into a small number of surgeons. These may include time, geographical or legal barriers of fewer surgeons providing care for patients from wider ranges of time and geography.

### Findings in context

The Lotka’s law, Pareto’s principle and Price’s law have been shown to approximate a wide variety of social phenomena both within (eg, length of hospital stay) and outwith healthcare (eg, population distributions),^1–8^ but it is not a universal phenomenon.^5 6^ This paper appears to demonstrate that monthly distribution of case volumes among spinal surgeons is another exception to these rules, and that the distribution among spinal units may be better approximated by alternatives (exponential decay in this case).

The concentration of case volumes is less than that described by Pareto and Price, which have been shown to hold in other fields. This may be reflective of the nature of the surgical industry. At the upper end, the impediment to higher volumes for each surgeon is the number of hours a surgeon is available to operate. A surgeon cannot operate perform more than one operation at once, nor perform operations in geographically disparate locations. These place a natural cap on the extent to which high output ‘superstars’ can dominate the output compared with a field such as academia (Price’s Law) or economics (the Pareto Principle). Similarly, the geography may be a factor that limits the ability of surgical units to dominate the output. There are also pressures at the lower end of the distribution, with calls for minimum case volumes.^22^ The casemix is another potential factor in case-volume concentration within spinal surgery. For example, some surgeons may spend a greater proportion of their practice to operating as compared with others. or a greater proportion of their practice to spinal operations. For others, spinal surgery may form only a part of their overall neurosurgical or orthopedic practice. In addition, some surgeons may be quicker at specific operations, whether that be due to experience, practice or talent. Finally, a small subset of individuals will undertake more complex operations that may take longer to complete. It was beyond the scope of this paper to investigate whether these findings would hold in a subgroup analysis where any impact of the case mix (such as elective vs emergency surgery, simple vs complex spine surgery) could be scrutinized and adjusted for.

**Figure 6:**
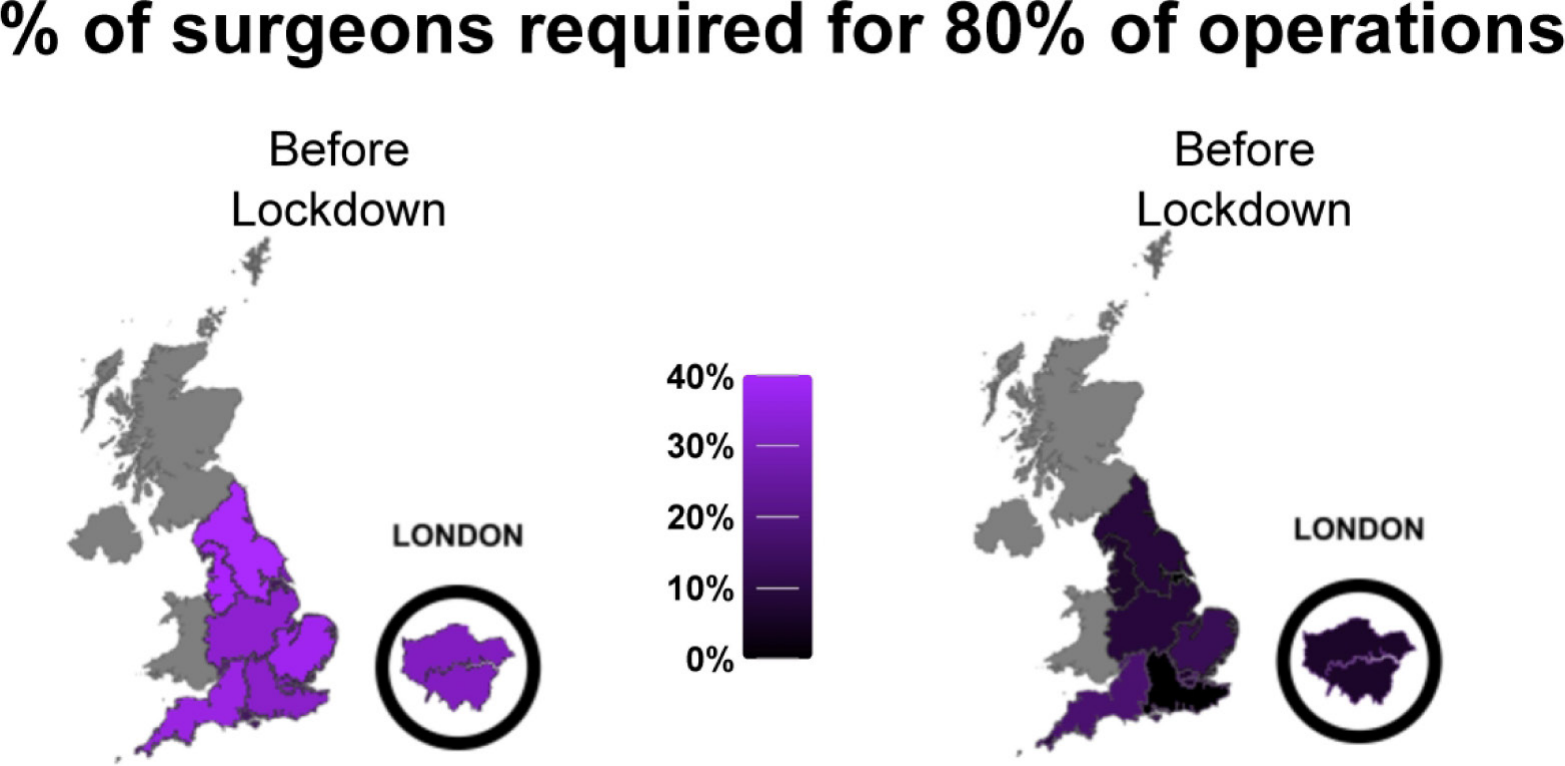
Geographical variations in case-volume concentration.

It is also difficult to be certain the extent to which the findings here would apply globally. One might reasonably expect that the general pattern in the distribution of cases may be similar in different global contexts, although the specific numerical values may differ, given that even within our data there were regional variations within a single nation (see figure 6). It may also be the case that in certain global regions, the pattern of distribution may be completely different—although we cannot make any judgment on this unless investigated further.

### Case volumes and outcomes

There has been a trend toward specialization in surgery over the last 40 years and also increase trend toward subspecialization within the specialty fields.^21^ There has been a trend toward specialization and subspecialization in surgery in modern times. This is supported by a multitude of studies showing that a surgeon’s case volume and extent of specialization is associated with improved patient outcomes.^23–26^ The extent of a surgeon’s specialization has been shown to be associated with improved outcomes in cranial and spinal disease independently of overall case volumes,^20^ and there has been a push toward reducing low-volume surgeons and increasing case-volume concentration into fewer specialist surgeons.

Despite this move toward increasing specialization, the extent to which the most productive surgeons dominate the total output of case volumes is less than would have been predicted from notable models derived from other industries such as the Pareto Principle or Price’s law. As discussed above, the nature of the surgical field may mean that a single surgeon is unable to dominate output in the same way that they might in other fields, whether this be due to constraints of time, geography or availability.

In addition, the consequences of workforce-level case-volume concentration on population-level outcomes is less clear. There is the theoretical risk of increased variation in population-level outcomes due to inequalities in access to specialist services. One study highlighted this risk in spinal surgery, where it was found that there was a lack of equipment, confidence and expertise in applying a halo vest for cervical spine trauma at district general hospitals.^27^ This suggests that there are potential negative population-level consequences of attempting a complete concentration of spinal case volumes into the hands of a few spinal subspecialist surgeons, particularly if some level of spinal surgical care by non-subspecialists proves inevitable.

### Strengths and limitations

We have, to our knowledge, given the first mathematical description of the distribution of output and specialization across a surgical (or medical) field on a national basis. We were able to derive objective measures to describe the extent of case volume inequality and service-level specialization that could be seen on inspection of the graphs.

The main limitation of this study is that while we have scrutinized the case volumes among the workforce, one must be cautious in using case volumes on an individual basis, as this would be confounded by the casemix of those individual surgeons. More complex highly specialist operations can take longer to complete than simple spinal operations, leading to lower number of completed operations. Although we did not possess the data to be able to stratify the analysis by the type of operation, this is ameliorated investigating workforce-level effects and trends. In addition, further studies are needed to establish the extent to which the results presented here are generalizable to other surgical or medical fields, or in other global regions (as discussed above). It may be that the coefficients of curves need adjusting, or different types of curves are needed altogether.

## Conclusions

While there is a reasonable degree of inequality in case volume distribution among spinal surgeons, this is less than in non-healthcare settings such as economics (Pareto’s principle) or scientometrics (Price’s law). The distribution among spinal units is closer to the aforementioned predictions by Pareto and Price. In addition, the distribution among surgeons do not follow the inverse power distribution (such as Lotka’s law) that was shown to hold true in many other areas, although the distribution among spinal units was again closer to the inverse power law. Given that increased surgeon-level case volumes is associated with improved patient-level outcomes, there is arguably scope for greater case-volume concentration on a workforce-level basis, although whether this would necessarily result in a population-level improvement would require further investigation.

## Data Availability

Data may be obtained from a third party and are not publicly available. Data were obtained from the British Spine Registry for a fee. Any requests for the data must be made to the British Spine Registry, or otherwise may only be release with their permission.

## References

[1] Hardy M . Pareto’s law. Math Intelligencer 2010;32:38–43. 10.1007/s00283-010-9159-2

[2] Nicholls PT . Price’s square root law: empirical validity and relation to Lotka’s law. Inf Process Manage 1988;24:469–77. 10.1016/0306-4573(88)90049-0

[3] Nicholls PT . Empirical validation of Lotka’s law. Inf Process Manage 1986;22:417–9. 10.1016/0306-4573(86)90076-2

[4] Pao ML . An empirical examination of Lotka's law. J Am Soc Inf Sci 1986;37:26–33. 10.1002/asi.4630370105

[5] Newman MEJ . Power laws, Pareto distributions and Zipf's law. Contemporary Physics 2005;46:323–51. 10.1080/00107510500052444

[6] Ijiri Y , Simon HA . Interpretations of departures from the Pareto curve firm-size distributions. J Pol Econ 1974;82:315–31. 10.1086/260194

[7] Reyes-Santias F , Reboredo JC , de Assis EM , et al . Does length of hospital stay reflect power-law behavior? A Q-Weibull density approach. Physica A: Statistical Mechanics and Its Applications 2021;568:125618. 10.1016/j.physa.2020.125618

[8] Wong T , Zhang EJ , Elhajj AJ , et al . The power law in operating room management. J Med Syst 2021;45:92. 10.1007/s10916-021-01764-1 34494167

[9] Weiser TG , Haynes AB , Molina G , et al . Size and distribution of the global volume of surgery in 2012. Bull World Health Organ 2016;94:201–209F. 10.2471/BLT.15.159293 26966331 PMC4773932

[10] von Elm E , Altman DG , Egger M , et al . The strengthening the reporting of observational studies in epidemiology (STROBE) statement: guidelines for reporting observational studies. Lancet 2007;370:1453–7. 10.1016/S0140-6736(07)61602-X 18064739

[11] The British Spine Registry: home. Available: https://www.britishspineregistry.com/ [Accessed 16 Apr 2021].

[12] Habeebullah A , Rajgor HD , Gardner A , et al . The impact of a spinal best practice tariff on compliance with the British spine registry. Bone Jt Open 2021;2:198–201. 10.1302/2633-1462.23.BJO-2020-0182 33739139 PMC8009900

[13] R Core Team . R: A language and environment for statistical computing. R Foundation for Statistical Computing; 2018. Available: https://www.R-project.org/

[14] Pinheiro J , Bates D , DebRoy S , et al . nlme: Linear and nonlinear mixed effects models. 2021. Available: https://CRAN.R-project.org/package=nlme [Accessed 12 Apr 2021].

[15] Vrieze SI . Model selection and psychological theory: a discussion of the differences between the Akaike information criterion (AIC) and the Bayesian information criterion (BIC). Psychol Methods 2012;17:228–43. 10.1037/a0027127 22309957 PMC3366160

[16] Wickham H , Chang W , Henry L , et al . ggplot2: Create elegant data visualisations using the grammar of graphics. 2021. Available: https://CRAN.R-project.org/package=ggplot2 [Accessed 20 Jun 2021].

[17] Pebesma E , Bivand R , Racine E , et al . sf: Simple features for R. 2021. Available: https://CRAN.R-project.org/package=sf [Accessed 20 Jun 2021].

[18] Bivand R , Rundel C , Pebesma E , et al . rgeos: Interface to geometry engine - open source ('GEOS'). 2020. Available: https://CRAN.R-project.org/package=rgeos [Accessed 20 Jun 2021].

[19] Bivand R , Keitt T , Rowlingson B , et al . rgdal: Bindings for the “Geospatial” data abstraction library. 2021. Available: https://CRAN.R-project.org/package=rgdal [Accessed 20 Jun 2021].

[20] Open geography portalx. Available: https://geoportal.statistics.gov.uk/ [Accessed 20 Jun 2021].

[21] Chapman JR , Wang JC , Wiechert K . Learning from disasters: the COVID-19 fallout on spine care. Global Spine Journal 2020;10:509–11. 10.1177/2192568220927719 32677556 PMC7359674

[22] GIRFT - Getting It Right First Time . Orthopaedic elective surgery - guide to delivering perioperative ambulatory care for patients with hip and knee pain requiring joint replacement surgery. 2023. Available: https://gettingitrightfirsttime.co.uk/wp-content/uploads/2023/07/Ambulatory-Hip-and-Knee-Replacement-Guide-March-2023-FINAL-V1-1.pdf [Accessed 04 Feb 2024].

[23] McCutcheon BA , Hirshman BR , Gabel BC , et al . Impact of neurosurgeon specialization on patient outcomes for intracranial and spinal surgery: a retrospective analysis of the nationwide inpatient sample 1998–2009. J Neurosurg 2018;128:1578–88. 10.3171/2016.4.JNS152332 28777023

[24] Sahni NR , Dalton M , Cutler DM , et al . Surgeon specialization and operative mortality in United States: retrospective analysis. BMJ 2016;354:i3571. 10.1136/bmj.i3571 27444190 PMC4957587

[25] Borowski DW , Kelly SB , Bradburn DM , et al . Impact of surgeon volume and specialization on short-term outcomes in colorectal cancer surgery. Br J Surg 2007;94:880–9. 10.1002/bjs.5721 17410637

[26] Chowdhury MM , Dagash H , Pierro A . A systematic review of the impact of volume of surgery and specialization on patient outcome. Br J Surg 2007;94:145–61. 10.1002/bjs.5714 17256810

[27] Rethnam U , Cordell-Smith J , Sinha A . Specialisation of spinal services: consequences for Cervical trauma management in the District hospital. J Trauma Manage Outcomes 2007;1. 10.1186/1752-2897-1-6 PMC2241763 18271985

