# Supplementary material for: Distribution of case volumes in surgery: an analysis of the British Spine Registry
Source: BMJ Surg Interv Health Technol. 2024 Mar 22;6(1):e000202. doi: 10.1136/bmjsit-2023-000202 (PMC10961580; doi:10.1136/bmjsit-2023-000202)
Supplement: Supplementary data [file bmjsit-2023-000202supp001.pdf]

SUPPLEMENTS

Table of Contents

Supplementary Figure 1. Number of (A) cases, (B) surgeons, and (C) units logged on BSR over time....2
TABLES....3
Supplementary Table 1. Percentage of entries with consultant in charge entered....3
Supplementary Table 2. Percentage of entries with unit entered....3

Koh CH, *et al. BMJ Surg Interv Health Technologies* 2024; 6:e000202. doi: 10.1136/bmjsit-2023-000202

Supplementary Figure 1. Number of (A) cases, (B) surgeons, and (C) units logged on BSR over time.

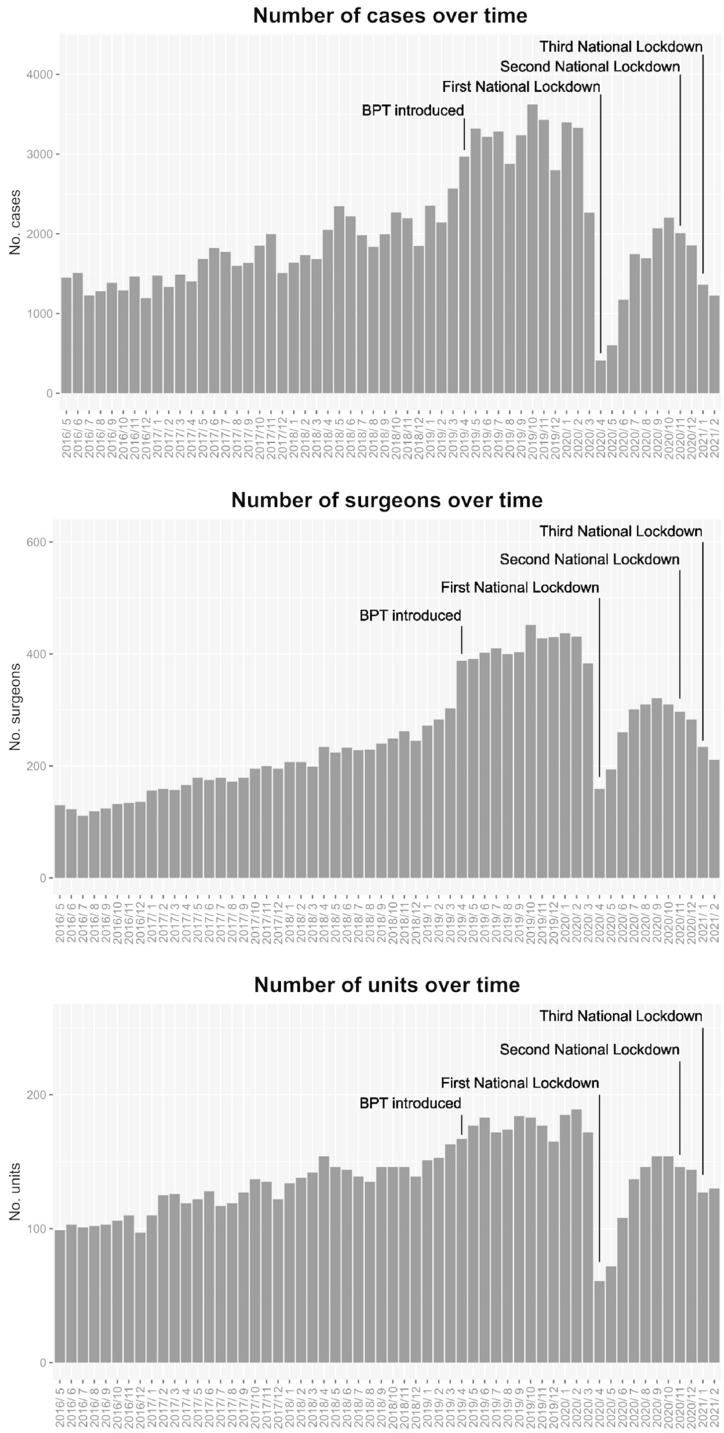

TABLES

Supplementary Table 1. Percentage of entries with consultant in charge entered.

		MONTH											
		**1**	**2**	**3**	**4**	**5**	**6**	**7**	**8**	**9**	**10**	**11**	**12**
**YEAR**	**2011**	50	33	32	36	31	50	53	60	57	47	48	33
	**2012**	33	54	45	21	25	16	3	6	4	4	2	4
	**2013**	3	4	5	4	4	3	3	3	6	4	3	3
	**2014**	3	3	4	2	3	4	5	3	5	5	4	5
	**2015**	5	6	6	5	7	6	6	6	7	6	6	5
	**2016**	8	9	14	42	88	89	86	88	86	87	89	92
	**2017**	90	87	94	94	93	94	95	96	96	96	93	93
	**2018**	91	92	92	92	92	94	91	93	94	95	95	96
	**2019**	98	95	95	92	93	95	95	96	97	100	100	100
	**2020**	100	100	100	100	100	100	100	100	100	100	100	100
	**2021**	100	100										

Supplementary Table 2. Percentage of entries with unit entered.

		MONTH											
		**1**	**2**	**3**	**4**	**5**	**6**	**7**	**8**	**9**	**10**	**11**	**12**
**YEAR**	**2011**	62	83	95	91	88	93	93	90	79	88	81	83
	**2012**	78	100	85	83	95	83	78	83	87	86	89	93
	**2013**	87	86	90	89	94	90	90	88	89	88	84	82
	**2014**	85	87	83	84	91	82	86	86	84	83	86	87
	**2015**	89	89	90	86	88	87	85	81	82	81	85	81
	**2016**	83	88	86	88	95	94	91	93	93	92	94	94
	**2017**	93	95	100	100	100	100	100	100	100	100	100	100
	**2018**	100	100	100	100	100	100	100	100	100	100	100	100
	**2019**	100	100	100	100	100	100	100	100	100	100	100	100
	**2020**	100	100	100	100	100	100	100	100	100	100	100	100
	**2021**	100	100										

Koh CH, *et al. BMJ Surg Interv Health Technologies* 2024; 6:e000202. doi: 10.1136/bmjsit-2023-000202
